# Descriptive epidemiological study of rare, less common and common cancers in Western Australia

**DOI:** 10.1186/s12885-021-08501-4

**Published:** 2021-07-08

**Authors:** Gemma A. Bilkey, Richard W. Trevithick, Emily P. Coles, Jennifer Girschik, Kristen J. Nowak

**Affiliations:** 1 Patient Safety and Clinical Quality, Clinical Excellence Division, Department of Health, East Perth, Western Australia 6004 Australia; 2 Office of Population Health Genomics, Public and Aboriginal Health Division, Department of Health, East Perth, Western Australia 6004 Australia; 3 Western Australian Cancer Registry, Clinical Excellence Division, Department of Health, East Perth, Western Australia 6004 Australia; 4 Epidemiology Branch, Public and Aboriginal Health Division, Department of Health, East Perth, Western Australia 6004 Australia

**Keywords:** Rare cancer, Less common cancer, Western Australia, Incidence, Survival, Epidemiology, RARECARE

## Abstract

**Background:**

There are no epidemiological studies describing rare cancers in Western Australia (WA). We aimed to fill this gap by estimating the incidence and five-year survival of rare, less common and common cancers in WA, based on definitions for rarity used by the Australian Institute of Health and Welfare and cancer groupings from the project on Surveillance of Rare Cancers in Europe (RARECARE). This research will enable policy- and decision-makers to better understand the size and nature of the public health problem presented by rare cancers in WA. It is anticipated that this study will inform improved health service design and delivery for all WA cancer patients, but particularly those with rare and less common cancers.

**Methods:**

We estimated incidence and five-year survival rates of rare, less common and common cancers in WA using data sourced from the WA Cancer Registry for the 2013–2017 period. Cancers were defined as rare (< 6), less common (6–12), or common (> 12) based on their crude incidence rate per 100,000 people per year.

**Results:**

Rare cancers make up 21.5% of all cancer diagnoses in WA, with a significantly poorer five-year survival of 58.2% (95% confidence interval (CI) 57.3–59.1%), compared to patients diagnosed with a common cancer, whose five-year survival was 87.8% (95% CI 87.3–88.3%). Survival for less common cancers was significantly poorer than both rare and common cancers, at 48.1% (95% CI 47.3–49.0%). Together, rare and less common cancers represent 48.4% of all cancer diagnoses in WA.

**Conclusions:**

While rare cancers are individually scarce, collectively over one in five cancer patients in WA are diagnosed with a rare cancer. These patients experience significantly worse prognoses compared to patients with common cancers.

**Supplementary Information:**

The online version contains supplementary material available at 10.1186/s12885-021-08501-4.

## Background

To date, there have been no studies investigating the descriptive epidemiology of rare cancers in Western Australia (WA) compared to less common and common cancers. The purpose of this study is to provide an overview of the rare cancer landscape in WA and support researchers, clinicians and policy makers to better understand areas of need and how best to support Western Australians living with rare cancers. The study will also contribute towards Priority 3.1 of the Australian *National Strategic Action Plan for Rare Diseases*, which seeks to use rare disease data to inform ‘care management, research and health system planning’ [1].

### Classification

There is no universally-agreed definition for what constitutes a rare cancer. The project on Surveillance of Rare Cancers in Europe (RARECARE) uses a definition of < 6 incident cases per 100,000 people per year while the United States (US) National Cancer Institute uses a definition of < 15 incident cases per 100,000 people per year [2, 3].

However, using measures of frequency alone as basis for a definition of ‘rare’ has limitations. For example, difficulty diagnosing rare cancers may make counts unreliable, especially in low-to-middle income countries with poorer access to diagnostic tools. A frequency definition may also mean lists of rare cancers developed in one region are inconsistent with other regions with different demographic profiles and could lead to different cut-off points, further hampering comparability. Additionally, the methods used to identify individual cancer entities (e.g., topography and/or morphology) influence the frequency of unique cancers and therefore the number and type of cancers classified as rare.

The RARECARE project has attempted to overcome some of the issues of using frequency measure only, by defining a list of clinically distinct cancers that can be applied consistently across regions, in conjunction with measures of frequency. The RARECARE groupings have been utilised globally to independently examine the epidemiology of rare cancers [4–8].

The RARECARE cancer groupings use the International Classification of Diseases for Oncology (ICD-O) to define cancers according to their topography (anatomical site) and morphology (histological type) [9]. These cancer groupings were developed through extensive consultation with experts such as pathologists, oncologists, patient advocacy groups, and cancer epidemiologists across the European Union (EU), and aspire to group cancers into three tiers that are clinically meaningful (see Table 1 for example) [2, 7, 8].

**Table 1:** Epithelial Tumours of the Nasal Cavity and Sinuses

Tier	Name
**1**	**EPITHELIAL TUMOURS OF NASAL CAVITY AND SINUSES**
**2**	**Squamous cell carcinoma with variants of the nasal cavity and sinuses**
3	Adenosquamous carcinoma
3	Basaloid squamous cell carcinoma
3	NUT carcinoma
3	Papillary squamous cell carcinoma
3	Squamous carcinoma
3	Squamous cell carcinoma, adenoid
3	Squamous cell carcinoma spindle cell
3	Verrucous carcinoma
**2**	**Lymphoepithelial carcinoma of nasal cavity and sinuses**
**2**	**Undifferentiated carcinoma of nasal cavity and sinuses**
**2**	**Intestinal type adenocarcinoma of nasal cavity and sinuses**

Tier 1 represents families of tumours, which require similar clinical expertise, relevant to the organisation of health care services [2, 10]. These are further stratified into tier 2 tumours, which are considered clinically distinct, as well as tier 3 tumours, which correspond to the World Health Organization classifications for individual tumour entities [2, 10]. Based on these groupings, the RARECARE project determined that cancers with a tier 2 incidence of less than 6 cases per 100,000 people per year could be defined as rare.

The Australian Institute of Health and Welfare (AIHW) and the Australian Government initiative, Cancer Australia, share the RARECARE definition for rare cancers, but also define ‘less common’ cancers (those with an incidence of 6–12 cases per 100,000 population) and provide a definition for ‘common’ cancers as an incidence of > 12 cases per 100,000 population. Herein, we utilise the AIHW and Cancer Australia definition for rare, less common and common cancers for consistency with these Australian Government agencies.

### Epidemiology

Despite the difficulty in defining rare cancers, collectively these cancers have been estimated to account for 22–24% of cancers overall [2, 11–13]. In Australia, the AIHW has estimated the number of rare, less common and common cancer cases nationally using ICD-10 codes to classify cancers primarily based on tumour sites [14]. However, these data are not broken down by jurisdiction and there is currently no WA estimate of the proportion of rare cancers in the state and no understanding of the descriptive epidemiology of who may be affected. Without state-specific data, the WA health system is unable to plan rare cancer services according to need, nor benchmark outcomes of rare cancer patients to determine if services are comparable to other jurisdictions or international standards. Additionally, WA policy-makers and decision-makers are unable to quantify the size and nature of the public health problem that rare cancers present.

WA is a geographically large jurisdiction with a low population density outside the metropolitan area. Delivering health care services to people living in regional and remote areas is already recognised as a challenge, and this becomes an even greater challenge for diseases that are rare, difficult to diagnose or require complex care and follow-up [15]. In this context, WA-specific epidemiological data on rare cancers is important to fulfil the objectives of the *National Strategic Action Plan for Rare Diseases,* as well as the first step in working to improve the lives of people living with rare cancers in WA.

Patients with rare cancers share commonalities in their experience of late or incorrect diagnoses [16] and less effective treatment options overall compared to common cancers [17]. Other challenges for rare cancer patients are a lack of therapeutic options stemming from difficulty recruiting patients for clinical trials [10, 18]; difficulty accessing optimal care due to variation in expertise between geographically diverse centres [19]; and financial challenges for patients to access therapeutic options, as these options may not meet criteria for public funding eligibility [16, 20, 21]. Clinicians also experience difficulty in developing expertise and best practice guidelines for individual rare cancers [13]. Consequently, patients with rare cancers tend to experience poorer health outcomes including lower five-year survival compared to those with common cancers (47% compared to 65% respectively in Europe) [2]. The five-year relative survival for patients with rare cancers has also been shown to vary between countries, with Germany, Italy, Belgium and Iceland experiencing 55% five-year relative survival compared to less than 40% in Bulgaria, Slovakia and Lithuania [13].

The aims of this paper are to: 1) develop the first comprehensive list of clinically distinct rare, less common and common cancers for an Australian jurisdiction by defining unique cancers according to the RARECARE groupings and using the rarity definitions adopted by the AIHW [22], 2) describe the demographic characteristics of people with rare cancers, 3) compare the demographic characteristics of people with rare, less common and common cancers, and 4) estimate five-year survival from rare cancers in WA.

## Methods

Data was provided by the WA Cancer Registry (WACR) for all persons for all tumours recorded in the WACR from 1 January 2013 to 31 December 2017, totalling 122,402 cases. All malignant cases were retained, along with 138 selected tumours that were either benign or where it was uncertain whether they were benign or malignant. The 138 additional tumours were predominantly central nervous system tumours including 74 cases of *pilocytic astrocytoma,* 15 cases of *myxopapillary ependymoma*, 14 cases of *ganglioma not otherwise specified (NOS)* and 10 cases of *subependymoma* tumours. These exceptions to the otherwise malignant group of tumours were based on expert requests and consensus for the original RARECARE study and are included here for consistency [23]. All other non-malignant tumours were removed, as well as 12,262 cases of non-notifiable epithelial skin tumours considered incomplete by the WACR, leaving a cohort of 122,402 cancer cases.

Cancers were divided into cancer families (tier 1) and clinically distinct tumours (tier 2) based on the RARECARE cancer groupings, which use ICD-O-3 codes to define cancers according to topography and morphology. In total there are 68 tier 1 and 216 tier 2 cancers mapped to ICD-O-3 codes defined by the RARECARE project as of February 2019. The “EPITHELIAL TUMOURS OF THE SKIN” cancer family was excluded from our study, as this comprised basal cell carcinomas and squamous cell carcinomas of the skin, which are not reportable to the WACR. This reduced the number of tier 1 and tier 2 tumour groups to 67 and 214 respectively for the WA analysis.

### Incidence

During the period 1 January 2013 to 31 December 2017, 64,950 cases of cancer were newly diagnosed. Crude five-year incidence rates of tier 1 and 2 cancers were estimated by dividing the number of new cases during this period by the total person-years in the overall WA population (estimated using the Australian Bureau of Statistics (ABS) Estimated Resident Population for 2013–2017) [24]. Tier 2 cancers were classified as rare, less common, or common (incidence of < 6, 6–12 and > 12 cases per 100,000 per year respectively) based on their crude five-year incidence rate. The crude incidence of sex-specific tier 2 cancers (i.e. those affecting only males or only females) were estimated separately for males and females, using the relevant sex-specific population denominators from the ABS Estimated Resident Population for 2013–2017. Age standardised incidence rates were also determined using the 2001 ABS Australian Standard Population [24].

#### Descriptive epidemiology

Incident cases of rare, less common, and common tier 2 cancers in WA were determined and stratified by sex, age, residential remoteness, socioeconomic status and Aboriginality for the 2013–2017 period.

Residential remoteness was classified by grouping ABS remoteness classifications into remote (combining very remote and remote), regional (combining outer and inner regional) or major city, based on each person’s residential postcode at the time of their diagnosis [25].

Socioeconomic status was determined by the ABS Index of Relative Socioeconomic Disadvantage (IRSD). IRSD is based on the collective characteristics of disadvantage for a geographical area, such as occupational skill level, education, and income [26]. The IRSD scores were assigned based on each person’s residential postcode at the time of their cancer diagnosis and were grouped into quintiles (with 1 being the most disadvantaged, and 5 the least disadvantaged).

The WACR collects information on whether a WA cancer patient is of Aboriginal or Torres Strait Islander descent (hereafter collectively referred to as Aboriginal in deference to Aboriginal peoples being the original custodians of WA). This information was used to determine Aboriginality for WA cancer cases from 2013 to 2017.

### Survival

Five-year observed and relative survival was estimated for each of tier 2 cancers using a period cohort methodology and a period window from 01 January 2013 to 31 December 2017.

Relative survival is the ratio of observed survival for cancer patients compared to expected survival in the underlying population, which accounts for deaths from causes other than cancer in the general population. The Ederer II method was used to calculate expected survival using the ABS 2013–2017 life table for WA [27, 28]. Of the 122,402 cases for the survival analysis 1712 cases were excluded as death certificate only (DCO) cases (*n* = 1, 044) or cases with the same diagnosis and death dates (*n* = 668). An additional 14,937 cases were removed for people that either died before or on entering the period window 1 January 2013 or that were diagnosed on the last day of the period window 31 December 2017. This resulted in a period survival cohort of 105,753 cases. Five-year relative survival was also estimated for rare, less common and common cancers broken down by sex, age, remoteness, and socioeconomic status.

### Data quality

The ability to identify the morphology and topography of a cancer to be accurately classified in cancer registries is duly limited by the nature of cancer. That is, patients may opt not to undergo further investigation, or there is genuine difficulty in reaching a precise diagnosis [29]. Cases with non-descriptive morphology codes such as *Neoplasm Not Otherwise Specified (NOS), Carcinoma NOS*, and *Non-Hodgkin Lymphoma NOS* may lead to the underestimation of the incidence of rare cancer entities as they are unable to be assigned to a specific (rare) cancer type [29].

Our results for NOS tumours compare favourably with other registries. There were 3.9% NOS cases for solid cancers (morphology codes: M8000–8001, M8800–8801) which was comparable to a similar study of US and EU cancer registries, which had figures of 3.2 and 9.6% of respectively [30]. Haematological cancers NOS (morphology codes: M9590–9591, M9760, M9800–9801, M9820 and M9860) were also comparable to the US and EU cancer registries, comprising 8.6% of cases compared to the US and EU proportions of 7.1 and 13.5% respectively [30]. The 0.1% of topography NOS cases were also comparable to international studies, such as the RARECARE study, which had 0.7% cases of topography NOS cases [2].

Of the 122,402 cancer cases, there were 0.8% DCO cases for WA which was indicative of good case-finding and/or traceback procedures for identifying earlier pathological, radiological, or clinical diagnoses [31], and was lower than the 3.0% DCO cases reported for the RARECARE project (Table 2) [2]. Additionally, 93.2% of cases were microscopically verified, suggestive of good diagnostic precision for cases in the cancer cohort. There were 0.1% of cases incidentally identified by autopsy which limited the impact these cases had on overall incidence estimates [31].

**Table 2:** Data quality indicators for WA cancers cases from 01 January 2013 to 31 December 2017

Total cases	DCO	Autopsy	Microscopic Verification	Solid Cancer NOS^**a**^	Haematological Cancer NOS^**b**^	Topography NOS^**c**^
122,402	1044 (0.8%)	99 (0.1%)	114,034 (93.2%)	4127 (3.9%)	971 (8.6%)	67 (0.1%)

^a^ Solid Cancer NOS morphology codes (8000, 8001, 8800, 8801)

^b^ Haematological Cancer NOS morphology codes (9590, 9591, 9760, 9800, 9801, 9820, 9860)

^c^ Topography NOS codes (C260, C268, C269, C390, C398, C399, C559, C579, C639, C689, C729, C759, C765, C767, C768)

These results lead us to conclude that the data is of reasonable quality and should produce reliable estimates of rare, less common and common cancers for WA.

## Results

### Incidence

#### Descriptive epidemiology of people with rare cancers in WA

Between 2013 and 2017, there were 13,995 rare cancer diagnoses in WA (Table 3). Males accounted for 57.8% (*n* = 8085) of diagnoses. The 65- ≤ 75 years age group represented the highest proportion of all rare cancer diagnoses, with 23.8% (*n* = 3325) of all rare cancer diagnoses for 2013–2017. The 0- ≤ 20 years age group represented the smallest number of rare cancer diagnoses overall, at 3.4% (*n* = 476).

**Table 3:** Incident cases and percentages of rare cancers by sex, age, remoteness, socioeconomic and Aboriginal status

	Rare
**Total**	13,995
**Sex**	
Male	8085 (57.8%)
Female	5910 (42.2%)
**Age (years)**	
0- ≤ 20	476 (3.4%)
20- ≤ 35	872 (6.2%)
35- ≤ 45	924 (6.6%)
45- ≤ 55	1749 (12.5%)
55- ≤ 65	2871 (20.5%)
65- ≤ 75	3325 (23.8%)
75- ≤ 85	2624 (18.7%)
> 85	1154 (8.2%)
**Remoteness**	
Major Cities	10,533 (75.3%)
Regional	2686 (19.2%)
Remote	772 (5.5%)
Not mapped	< 5* (< 1%)
**IRSD quintile**	
1- Most Disadvantaged	1856 (13.3%)
2	3819 (27.3%)
3	2577 (18.4%)
4	2267 (16.2%)
5 – Least Disadvantaged	3469 (24.8%)
Not mapped	7 (< 1%)
**Aboriginality**	
Aboriginal	365 (2.6%)
Non-Aboriginal	13,554 (96.8%)
Not mapped	76 (< 1%)

With respect to residential address at time of diagnosis, 75.3% of all rare cancer diagnoses were for patients from major cities. Additionally, 13.3% of all rare cancer diagnoses were from the most disadvantaged socioeconomic quintile, with 24.8% from the least disadvantaged quintile. Quintile 2 represented the majority of rare cancer diagnoses, accounting for 27.3% of all rare cases diagnosed. While it is estimated that 3.9% of Western Australians identify as Aboriginal [32], Aboriginal people represented 2.6% of all rare cancer diagnoses.

#### Demographic characteristics of people with rare cancers compared with less common and common cancers

Overall, there were 64,950 cases cancer diagnosed from 2013 to 2017. Of these, 21.5% were rare, 26.9% were less common and 51.6% were common at the tier 2 level (Table 4).

**Table 4:** Incident cases and percentages of cancers by rarity, sex, age, remoteness, socioeconomic and Aboriginal status

	Rare	Less Common	Common
**Total**	13,995 (21.5%)	17,462 (26.9%)	33,493 (51.6%)
**Sex**			
Male	8085 (22.4%)	9714 (27.0%)	18,245 (50.6%)
Female	5910 (20.4%)	7748 (26.8%)	15,248 (52.8%)
**Age (years)**			
0- ≤ 20	476 (77.2%)	114 (18.5%)	25 (4.1%)
20- ≤ 35	872 (45.2%)	443 (23.0%)	614 (31.8%)
35- ≤ 45	924 (27.1%)	729 (21.4%)	1753 (51.5%)
45- ≤ 55	1749 (21.6%)	1756 (21.6%)	4608 (56.8%)
55- ≤ 65	2871 (19.9%)	3132 (21.7%)	8428 (58.4%)
65- ≤ 75	3325 (18.5%)	4406 (24.5%)	10,268 (57.0%)
75- ≤ 85	2624 (20.6%)	4194 (32.9%)	5938 (46.6%)
> 85	1154 (20.2%)	2688 (47.1%)	1859 (32.6%)
**Remoteness**			
Major Cities	10,533 (21.3%)	13,470 (27.2%)	25,516 (51.5%)
Regional	2686 (21.6%)	3186 (25.7%)	6543 (52.7%)
Remote	772 (25.7%)	804 (26.8%)	1429 (47.6%)
Not mapped	< 5^a^	< 5^a^	5
**IRSD quintile**			
1- Most Disadvantaged	1856 (23.5%)	2329 (29.5%)	3722 (47.1%)
2	3819 (22.0%)	4706 (27.1%)	8818 (50.8%)
3	2577 (21.7%)	3280 (27.7%)	6001 (50.6%)
4	2267 (22.5%)	2575 (25.6%)	5225 (51.9%)
5 – Least Disadvantaged	3469 (19.5%)	4568 (25.7%)	9715 (54.7%)
Not mapped	7	< 5^a^	12
**Aboriginality**			
Aboriginal	365 (38.3%)	290 (30.5%)	297 (31.2%)
Non-Aboriginal	13,554 (21.3%)	17,116 (26.9%)	33,038 (51.9%)
Not mapped	76 (26.2%)	56 (19.3%)	158 (54.5%)

*IRSD* Index of Relative Socio-economic Disadvantage; ^a^ < 5: Suppressed case numbers as cases < 5 [33]

The demographic profiles comparing rare, less common and common cancers considering sex, age, remoteness and socioeconomic status were similar across the three groups.

However, Aboriginal people with cancer experienced a greater proportion of rare cancers than the non-Aboriginal population (38.3% versus 21.3% of rare cancer cases respectively).

#### Survival

**Fig. 1:**
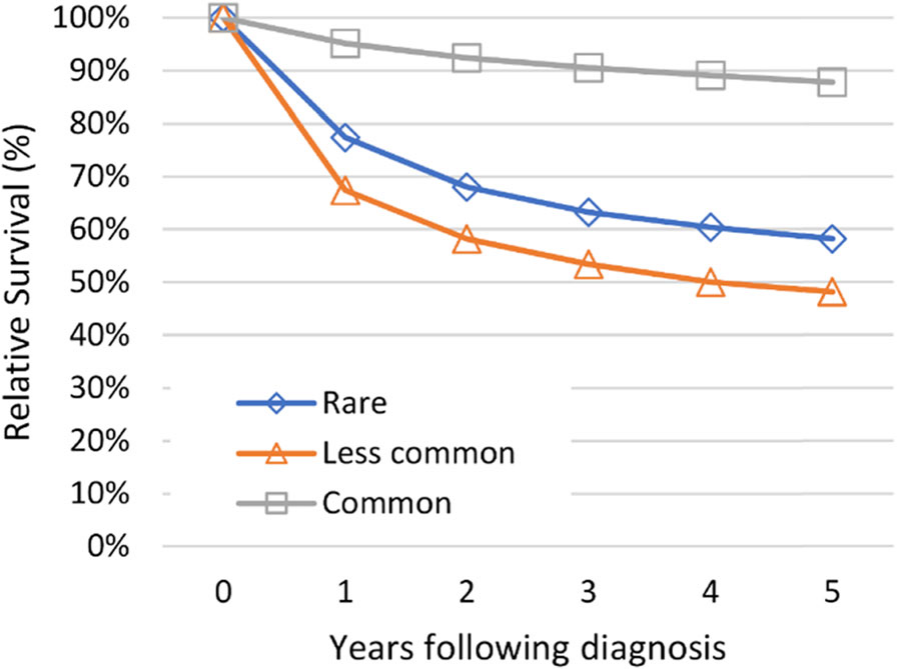
Five-year relative survival for patients with rare, less common and common cancers

The five-year relative survival rate for rare cancers was 58.2% (95% confidence interval (CI) 57.3–59.1%). This is compared to a five-year relative survival for common cancers of 87.8% (95% CI 87.3–88.3%). However, five-year relative survival for less common cancer was significantly poorer than both rare and common cancers, at 48.1% (95% CI 47.3–49.0%) (Fig. 1).

**Fig. 2:**
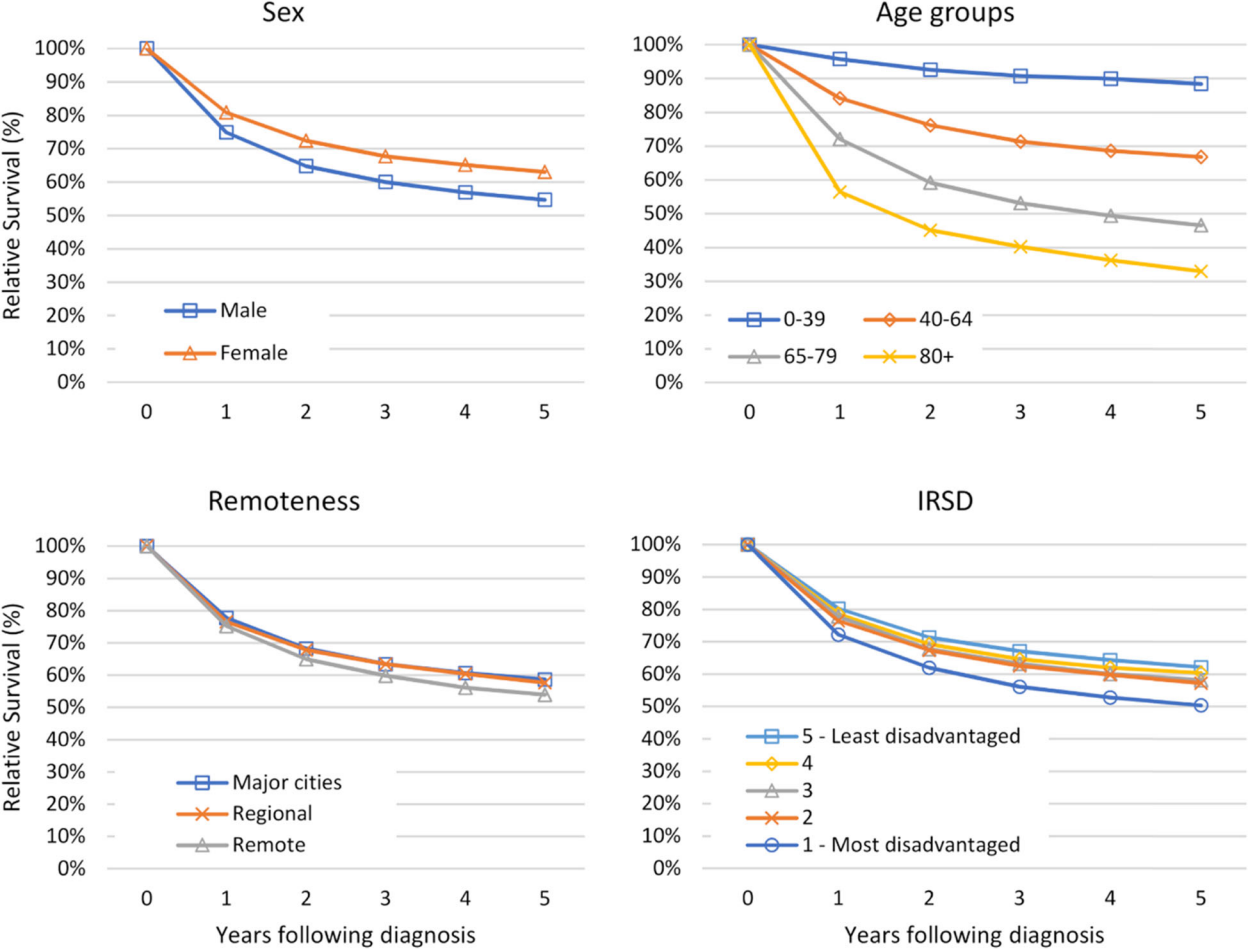
Five-year relative survival for rare cancers by sex, age-group, remoteness and IRSD

Females diagnosed with a rare cancer experienced significantly better five-year relative survival than males, at 63.0% (95% CI 61.6–64.4%) compared to 54.7% (95% CI 53.4–55.9%). Five-year relative survival also decreased with increasing age-group at diagnosis (Fig. 2).

Five-year relative survival was worse overall for rare cancer patients who lived in a remote area at the time of diagnosis (53.9, 95% CI 50.0–57.7%) compared to those who lived in a major city (58.7, 95% CI 57.6–59.7%) (Fig. 2).

People diagnosed with a rare cancer in the most disadvantaged quintile experienced significantly poorer five-year relative survival than those in the least disadvantaged quintile, at 50.4% (95% CI 47.8–52.8) compared to 62.2% (95% CI 60.3–64.0) (Fig. 2) A complete list of the relative survival estimates and confidence intervals for each of the comparisons (cancer rarity, sex, age-group, remoteness and IRSD) are provided in an additional file (Additional file 1).

#### List of rare, less common and common tier 2 cancers for WA

The five most common rare cancers were *poorly differentiated endocrine carcinoma of lung*, *squamous cell carcinoma with variants of cervix uteri, squamous cell carcinoma with variants of lip, Hepatocellular carcinoma of liver and IBT,* and *well differentiated not functioning endocrine carcinoma of pancreas and digestive tract.* The full list of rare, less common and common cancers, including incidence and five-year survival estimates, are included in an additional file (see Additional file 2).

Common cancers for the overall WA population included *malignant skin melanoma*, *adenocarcinoma with variants of the prostate*, and *renal cell carcinoma with variants*. Less common cancers included *adenocarcinoma with variants of the pancreas*, *transitional cell carcinoma of the bladder*, and *astrocystic tumours of CNS*.

For confidentiality, cells with fewer than five cases have been suppressed [33]. Additionally, age-standardised rates are only presented for cancers with 20 or more cases as per statistical guidelines [34].

## Discussion

This study is the first of its kind to provide descriptive epidemiology of rare cancers in WA and the first comprehensive list of clinically distinct rare, less common and common cancers for an Australian jurisdiction. While another Western Australian study has considered the number of hospital admissions attributable to rare diseases, including rare cancers [35], never before has a study used WACR data to determine all cancer types that are rare for Western Australians. Our comprehensive and contemporary list of rare, less common, and common cancers will enable more targeted health service planning for cancer patients, benefitting current and future patients with cancer, as well as the broader health system and population.

Our results reinforce an overall sex differential in survival in favour of females found in a previous Australian study of Victorian cancer registry data of 25 select cancers [35]. Aboriginal people are known to experience worse cancer outcomes, such as poorer survival (45), with contributing factors including lower rates of education and employment, higher rates of smoking, and difficulty accessing the specialist health care services needed for the diagnosis and management of cancer (13, 46, 47). Our results suggest that Aboriginal people with a cancer diagnosis are also more likely to be diagnosed with a rare cancer than non-Aboriginal people. Further analysis of this data is warranted to understand whether there is a true difference in the proportion of rare cancers diagnosed in Aboriginal people or whether outside factors have influenced the proportion of rare cancers for Aboriginal people diagnosed with cancer. Additional research, particularly at a national level, could also help to uncover whether cancers considered to be rare for the overall WA population are consistent with those that are rare among Aboriginal peoples.

The findings of our study support international findings that patients with rare cancer have a poorer five-year survival than patients diagnosed with a common cancer. As a group, rare cancers make up 21.5% of all cancers diagnosed in WA. Our results show close to a 30% higher mortality at 5 years for patients in WA diagnosed with a rare cancer compared with patients diagnosed with a common cancer.

Our results also show that patients from the most disadvantaged quintile have significantly poorer five-year relative survival compared to those from the least disadvantaged quintile. This supports previous studies that have found socioeconomic disadvantage is a predictor of patient cancer outcomes [36–38].

Compared to international data, the five-year relative survival of 58.2% for patients diagnosed with a rare cancer in WA is similar to the 55% experienced in Germany, Italy, Belgium and Iceland, and was notably better than the 40% five-year relative survival experienced in Bulgaria, Lithuania and Slovakia [13]. Of note, our data uncovered that the poorest five-year relative survival was experienced by patients diagnosed with a less common cancer, with an almost 40% higher mortality at 5 years compared to patients diagnosed with a common cancer. It is proposed that this discrepancy is because pancreatic cancer falls into the less common category, which is known to have very poor survival outcomes [14]. Collectively, rare and less common cancers represent almost half of all cancers diagnosed in WA, which aligns with the United Kingdom where rare and less common cancers were found to represent 47% of all cancer diagnoses [39]. As such, the significantly poorer prognosis applies to almost 1 in 2 people diagnosed with cancer in WA.

The lower five-year survival rates for rare cancer patients in remote areas relative to major cities was in keeping with a recent international meta-analysis and review that revealed poorer survival in rural patients compared to metropolitan patients [40].

Our study results support international findings that survival for patients diagnosed with rare cancer declines with advancing age of diagnosis [5, 41]. While age is key factor in survival, it also influences opportunities for research, as the elderly are often excluded from clinical trials [13]. Furthermore, older patients may have poorer survival due to lower application of standard treatment protocols due to more advanced stage at diagnosis, such protocols not being tailored for the elderly, or survival may be adversely affected due to more complications from comorbidities compared with younger patients [36, 41].

Cancer classification systems have evolved from being based solely on anatomical location, to the use of microscopic examination of cell types over 100 years ago to guide diagnosis and management, to the current use of molecular profiles and genomics to distinguish the individual features of each patient’s cancer [18]. In its extreme, this latter method is likely to render every diagnosis of cancer a rare entity by definition in the future [18]. However, while molecular profiles influence treatment choice, they are not yet utilised to determine the prognosis or natural history of cancer types [37]. Until this time, cancer lists such as the RARECARE list used in our study will continue to be an important mechanism for policy- and decision-makers to understand and address the gap in prognosis for patients diagnosed with a rare and less common cancers in WA. For consideration by policy- and decision-makers, is the European finding that rare and less common incidence rates are increasing, possibly as a result of better technologies to diagnose and describe these less common entities [2]. This leads us to challenge decision-makers to address this unmet clinical need for patients diagnosed with both rare and less common cancers in WA.

### Strengths and limitations

The classification of rare cancers may not reflect the true proportion of rare cancers in the population due to the difficulty in diagnosing rare cancers [29]. It is also difficult to derive stable and accurate estimates for rare cancer incidence and survival in a setting of low case numbers [38]. A strength of our study stems from the legislative requirement in WA that all cancer diagnoses are reported to the WACR [42], which minimises count underestimates by providing a comprehensive snapshot of cancer cases diagnosed in the WA population each year. Furthermore, the accepted ‘best criterion for a definition of rare cancer is incidence, rather than prevalence’ [9] as the sub-acute nature of most cancers means prevalence is likely to assign rarity to cancers with poor prognosis even when they are common and vice versa [2].

Discordance in complex diagnoses is also a challenge for rare cancers, even with recent advances in diagnostic tools, such as ongoing disagreement in the categorisation of sarcomas between pathologists and expert panels [43]. Additionally, cancer stage at diagnosis, which could be used to improve incidence estimates, is not collected in the WACR and was not available for analysis.

Determination of Aboriginal status in the WACR is based on identification in primary data sources, such as pathology forms and hospital morbidity records. This may under-represent the true proportion of Aboriginal people in the dataset. Future analyses could improve upon this study by using linked data to validate whether cancer record/s belong to an Aboriginal person. Future analyses would also benefit from life tables for Aboriginal peoples for a range of time periods, which would enable five-year relative survival to be determined.

### Future research opportunity

Further exploration to understand the disparities in rates of rare cancers for Aboriginal and non-Aboriginal Western Australians would also better inform health service design for Aboriginal people. In addition, this research provides a benchmark to explore benefits that advances in genomic and molecular sequencing of cancers, surveillance programs for known genetic risks, liquid biopsies for cancer detection, and precision oncology treatments may have on survival and outcomes for patients with rare cancer in WA.

## Conclusions

This study provides the first comprehensive list of clinically distinct rare, less common and common cancers for an Australian jurisdiction. Rare and less common cancers make up 21.5 and 26.9% of all cancers diagnosed in WA respectively. The five-year cumulative relative survival for rare cancers is 58.2% compared with 48.1% for patients diagnosed with less common cancers and 87.8% for patients diagnosed with common cancers, demonstrating the poorer outcomes experienced by patients diagnosed with rare and less common cancers in WA.

## Supplementary Information

**Additional file 1: Supplementary Appendix 1.** Measures of precision: confidence intervals for 5-year relative survival estimates. Five-year relative-survival estimates and confidence intervals for rare, less common and common cancers, as well as rare cancers (broken down by sex, age-groups, remoteness, and IRSD) for Western Australia.

**Additional file 2: Supplementary Appendix 2.** Incidence and five-year survival for cancers in Western Australia. List of rare, less common and common cancers, including incidence and five-year survival estimates, for Western Australia.

## Data Availability

The data generated and/or analysed during the current study are not publicly available due to data access and analysis requiring ethics approval from the Western Australian Department of Health Human Research Ethics Committee and WA Aboriginal Health Ethics Committee. Researchers may request the data analysed during this study from the WA Cancer Registry (wacanreg@health.wa.gov.au) or contacting the corresponding author on reasonable request.

## Abbreviations

ABS: Australian Bureau of Statistics
AIHW: Australian Institute of Health and Welfare
CI: Confidence Interval
DCO: Death Certificate Only
EU: European Union
IRSD: Index of Relative Socioeconomic Disadvantage
NOS: Not Otherwise Specified
RARECARE: Project on Surveillance of Rare Cancers in Europe
US: United States
WA: Western Australia
WACR: WA Cancer Registry
