# Supplementary material for: Descriptive epidemiological study of rare, less common and common cancers in Western Australia
Source: BMC Cancer. 2021 Jul 8;21:779. doi: 10.1186/s12885-021-08501-4 (PMC8265087; doi:10.1186/s12885-021-08501-4)
Supplement: Supplementary file 1 — Additional file 1: Supplementary Appendix 1. Measures of precision: confidence intervals for 5-year relative survival estimates. Five-year relative-survival estimates and confidence intervals for rare, less common and common cancers, as well as rare cancers (broken down by sex, age-groups, remoteness, and IRSD) for Western Australia. [file 12885_2021_8501_MOESM1_ESM.docx]

**SUPPLEMENTARY APPENDIX 1: Measures of precision: confidence intervals for 5-year relative survival estimates**


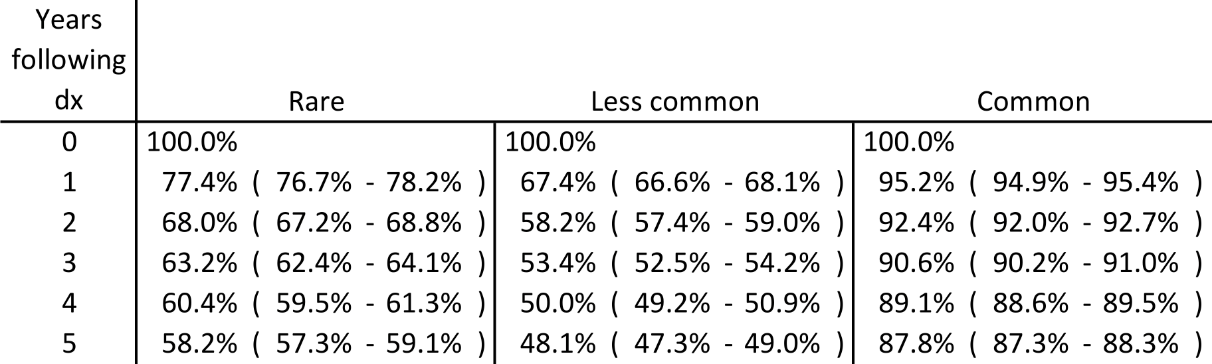


**Figure 1.** Five-year relative survival for rare, less common and common cancers.


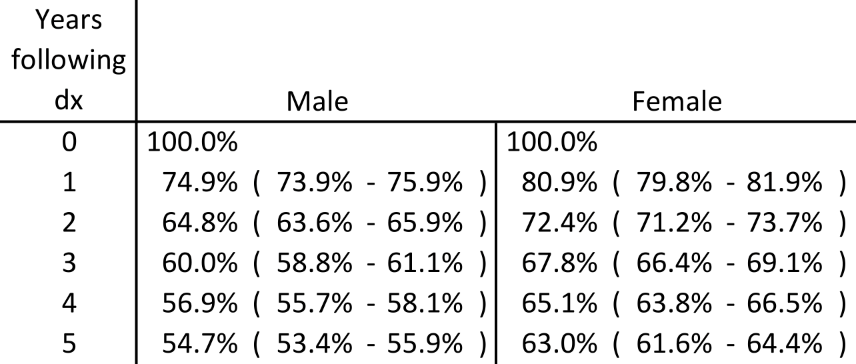


**Figure 2.** Five-year relative survival for rare cancers by sex.


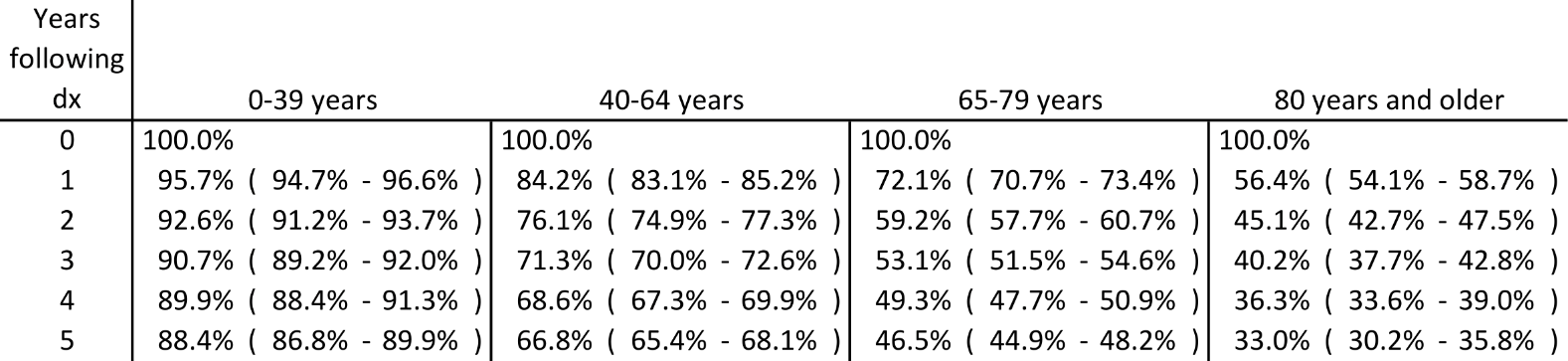


**Figure 3.** Five-year relative survival for rare cancers by age groups.


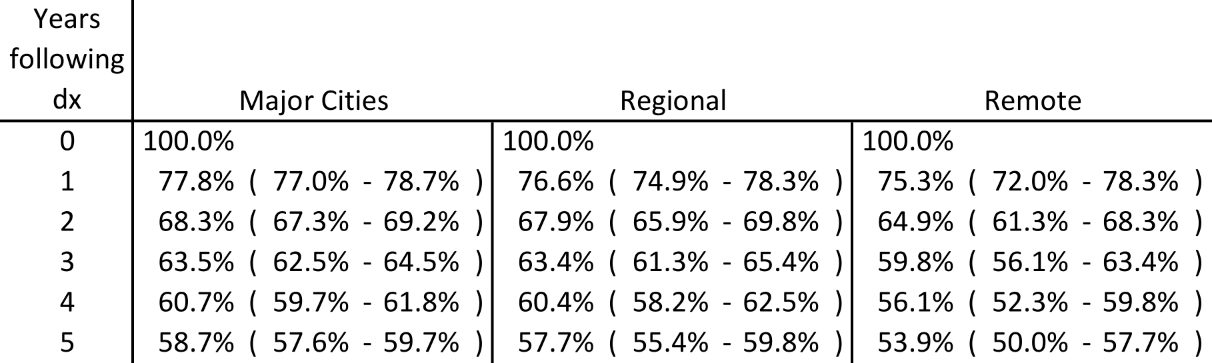


**Figure 4.** Five-year relative survival for rare cancers by remoteness.


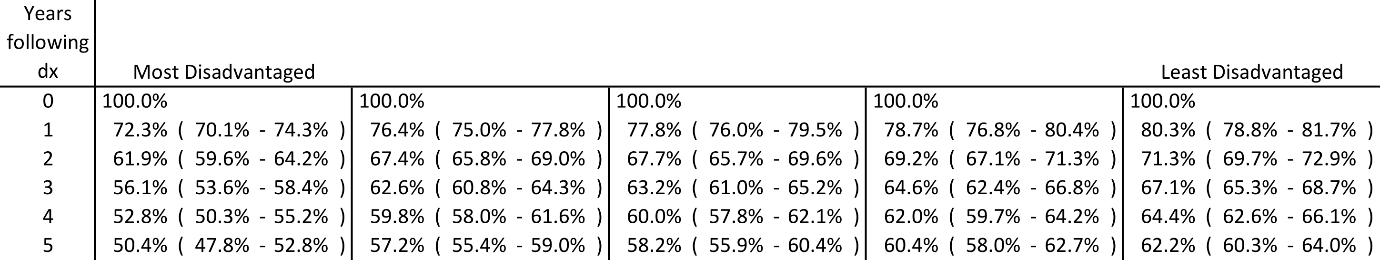


**Figure 5.** Five-year relative survival for rare cancers by Index of Relative Social Disadvantage (IRSD).
